# Genomic Identification and Comprehensive Characterization of the CAMTA Transcription Factor Family in *Brassica rapa* L.

**DOI:** 10.1155/ijog/1676159

**Published:** 2026-07-11

**Authors:** Jannatul Afrin, Maria Chowdhory, Subroto Das Jyoti, Jaber Bin Azim, Nikunjo Chakroborty, Arif Hasan Khan Robin

**Affiliations:** ^1^ Department of Genetics and Plant Breeding, Bangladesh Agricultural University, Mymensingh, Bangladesh, bau.edu.bd; ^2^ Biotechnology Division, Bangladesh Rice Research Institute (BRRI), Gazipur, Bangladesh, brri.gov.bd; ^3^ Department of Soil and Crop Sciences, Texas A&M University, College Station, Texas, USA, tamu.edu

**Keywords:** *B. rapa*, *CAMTA* gene family, evolutionary linkage, expression profiling, structural stability

## Abstract

Calmodulin‐binding transcription activators (*CAMTAs*) are a key family of transcription factors that regulate gene expression involved in plant development and mediate plant responses to a wide range of biotic and abiotic stresses. Although *CAMTA* genes have been surveyed in several crop species, their comprehensive characterization in *Brassica rapa* remains limited. In this study, we performed a genome‐wide analysis and identified 12 *BrCAMTA* genes, dispersed across 10 chromosomes of the *B. rapa* genome. Phylogenetic analysis of 84 CAMTA proteins from seven plant species grouped them into four major clades, with BrCAMTAs exhibiting conserved exon–intron structures and motif compositions within their respective phylogenetic groups. Domain analysis revealed that the major CG‐1 domain is conserved across all the genes. Protein–protein interaction analysis indicated that the predicted interacting proteins contain diverse domains and exhibit a variety of functions. Promoter analysis revealed a wide array of cis‐regulatory elements linked to hormone signaling, stress responses, and developmental regulation. RNA‐seq–based expression profiling revealed tissue‐specific expression patterns, with *BrCAMTA* genes showing high expression in roots, stems, and callus tissues. Notably, *BrCAMTA6*, *BrCAMTA8*, and *BrCAMTA11* exhibited significantly higher expression across all examined tissues. Collectively, these findings highlight the structural conservation and functional diversity of *CAMTA* genes in *B. rapa* and provide a valuable framework for future validation studies that are aimed at understanding their roles in growth, development, and environmental adaptation.

## 1. Introduction

Plant development and adaptation to stress are intricately regulated by calmodulin‐binding transcription activators (*CAMTAs*), a class of Ca^2+^/CaM‐mediated transcription factors (TFs). Calcium ions (Ca^2+^) serve as ubiquitous secondary messengers that modulate numerous cellular pathways in eukaryotes, including plants [1, 2]. Alongside Ca^2+^‐dependent protein kinases (CDPKs), regulatory components such as calmodulin (CaM), CaM‐like proteins, and calcineurin B‐like proteins participate in Ca^2+^‐mediated transcriptional regulation at both the nuclear and cellular levels [3, 4]. CaM acts as a primary calcium sensor, binding to downstream proteins and TFs to initiate elicitor responses and modulate various physiological and developmental processes [5, 6].

TFs play a vital role in plant adaptation by integrating environmental cues to regulate the expression of numerous stress‐responsive genes [7]. *CaMs* and *CAMTAs* together have been reported to regulate over 90 TFs [8, 9]. CAMTAs contain conserved domains: CG‐1 (calmodulin‐binding transcription activator 1) for DNA binding, TIG (transcription factor immunoglobulin) for nonspecific DNA interaction, ANK (ankyrin repeats) for protein–protein interactions (PPIs), CaMBD (calmodulin‐binding domain), and IQ motifs (isoleucine‐glutamine motifs) for calmodulin binding [10, 11]. Nuclear localization signals (NLS) guide CAMTA proteins into the nucleus where they bind specific cis‐regulatory elements primarily (A/C/G) CGCG(T/C/G) or (A/C) CGTGT motifs in target gene promoters to regulate transcription [6, 12, 13].

CAMTAs were first discovered in tobacco [14] and, with the rise of next‐generation sequencing and bioinformatics tools, have since been characterized in numerous plant species. These include *Arabidopsis* (6 genes), rice (5), sesame (5), rapeseed (18), soybean (15), maize (11), poplar (8), citrus (10), and wheat (15) [8, 10, 11, 15–20]. Diploid plants like *Arabidopsis* generally have fewer *CAMTA* genes compared with polyploids like *Brassica napus* and soybean, which have experienced multiple genome duplications [21]. *CAMTA* genes are well established as regulators of plant responses to both abiotic and biotic stressors. In *Arabidopsis*, *CAMTA1* and *CAMTA3* mutants exhibit hypersensitivity to freezing temperatures [22]. *AtCAMTA3* contributes to cold tolerance by targeting conserved Motif 2 (CM2) in the CBF2 promoter, a key regulator of cold response. Similarly, *AtCAMTA1* is involved in drought tolerance by regulating genes such as *RD26*, *ERD7*, *LTPs*, *RAB18*, *COR78*, *HSPs*, and *CBF1* [23, 24].

Beyond model species, *CAMTAs* in crops show similar functional relevance. For instance, *BnCAMTA3* mutants in *B. napus* exhibit increased resistance to cold and pathogen attack [20]. In soybean, *GmCAMTA2* and *GmCAMTA8* coordinate circadian regulation during development and drought stress responses [25]. In tomato (*Solanum lycopersicum*), seven *SR/CAMTA* genes have been cloned, and their expression is modulated by ethylene signaling throughout fruit ripening and storage [26]. *SlSR1L* is upregulated during drought, and its suppression reduces drought tolerance [27]. In wheat, over 580 genes contain CGCG or CCGCGT cis‐elements in their promoters, which are potential *CAMTA* targets involved in processes such as RNA regulation, signal transduction, stress responses, hormone metabolism, and lipid metabolism [28].

Although CAMTAs have been widely investigated in various plant species, a comprehensive genome‐wide identification and characterization of this gene family has not yet been conducted in *B. rapa*, an important crop within the Brassicaceae family. *B. rapa* encompasses a wide range of morphotypes, including oilseed varieties, leafy vegetables, and root crops [29]. Given its agronomic significance and evolutionary relevance in the *Brassica* lineage, investigating the *CAMTA* gene family in *B. rapa* is both timely and necessary. This study is aimed at performing a genome‐wide identification of *CAMTA* genes in *B. rapa*, analyzing their structural features and phylogenetic relationships, identifying their regulatory elements, and evaluating their expression patterns across tissues. Our findings are aimed at uncovering candidate genes associated with growth and stress resilience, providing a valuable resource for further functional genomics and crop improvement efforts in *Brassica* species.

## 2. Materials and Methods

### 2.1. Identification of *CAMTA* Family Genes

We identified 12 *CAMTA* genes in *B. rapa* to perform a genome‐wide characterization of the *CAMTA* gene family from the BRAD database [30]. To find *CAMTA* genes throughout the *Brassica rapa* genome, we used CAMTA protein sequences from *Arabidopsis thaliana*, *Brassica oleracea*, *Glycine max* (soybean), and *Nicotiana tabacum* (tobacco). We further enhanced this search using the hidden Markov model (HMM) profile for the CG‐1 domain (PF03859), obtained from the Pfam database (https://pfam.xfam.org/), applying an E‐value threshold of < 1e‐5. Genomic sequences, coding DNA sequences (CDS), and protein sequences of *B. rapa* were obtained from the *Brassica* database (https://www.brassicadb.cn/; Versions 3.3.1 and 3.5; accessed June 10, 2022) (Data Files S1, S2, and S3). CAMTA protein sequences for *Arabidopsis* (6), rice (6), wheat (10), maize (7), tobacco (19), and soybean (24) (Data File S1) were collected from Ensembl plants [31], iTAK [32], MaizeGDB [33], and PlantTFDB databases [34].

### 2.2. Prediction of Physicochemical Properties

The physicochemical properties, including amino acid number (AA), molecular weight (MW), theoretical isoelectric point (pI), and grand average of hydropathicity (GRAVY), of the BrCAMTA proteins were analyzed using the ProtParam tool available on the ExPASy server (https://web.expasy.org/protparam/) [35]. Subcellular localization was predicted using Cell‐Ploc 2.0 [36]. The positions of CG‐1 domains in the 12 BrCAMTA proteins were identified using the Pfam database [37] and the SMART domain search tool [38]. Domain presence was further validated through the HMMER web server [39] and InterPro (https://www.ebi.ac.uk/interpro).

### 2.3. Phylogenetic Analysis, Synteny Analysis, and Multiple Sequence Alignment of BrCAMTA Proteins

The ClustalW program (Version 2.1) was explored to analyze the protein sequences of *CAMTA* family members in crops such as *B. rapa*, rice (*O. sativa*), *Arabidopsis* (*A. thaliana*), soybean (*G. max*), maize (*Z. mays*), wheat (*T. aestivum*), and tobacco plants (*N. tabacum*) [40]. To produce a dendrogram, MEGA X software is used for the CAMTA family protein sequences of crops like *B. rapa*, *Arabidopsis*, and other crops. This involved utilizing the neighbor joining method and conducting 1000 bootstrap replications [41]. The phylogenetic tree was edited using iTOL (Interactive Tree of Life), an online visualization tool available at https://itol.embl.de/ [42].

Synteny analysis was done using the MCScanX algorithm [43], and visualization of the results was performed using TBtools [44].

The alignment of protein sequences was accomplished using the Clustal X v2 tool for crops such as *B. rapa*, *Arabidopsis*, *B. oleracea*, and *Raphanus sativus* [45]. The results were then organized in GeneDoc following protocols [46].

### 2.4. Assessment of Structural Features

Gene structure information, comprising exons, CDS, and untranslated regions (UTRs), was analyzed from GFF3 files using TBtools software (v2.376) [44]. The GFF3 files were retrieved from the Phytozome database [47]. Domain‐related hit data were obtained from the NCBI Batch CD‐Search tool for domain analysis (https://www.ncbi.nlm.nih.gov/Structure/bwrpsb/bwrpsb.cgi). Conserved motifs within the BrCAMTA proteins were identified using the Simple MEME Wrapper function in TBtools. Finally, the visualization of gene structures, including motifs, domains, and exon–intron organization, was performed using the Gene Structure View (Advanced) module in TBtools (v2.376).

### 2.5. Chromosomal Locations, Gene Duplication, and Calculation of Ka/Ks Ratios

The genomic coordinates, including start and end sites, chromosome assignment, and gene length of the 12 *BrCAMTA* genes, were retrieved from the Ensembl Plants database [31]. Their precise chromosomal distribution in *B*. *rapa* was visualized using the MapGene2Chrom v2 web tool [48].

Gene duplication among BrCAMTA proteins was assessed using NCBI BLAST by comparing the percentage of query coverage between the genes [49].

Using the TBtools, the number of synonymous (Ks) and nonsynonymous (Ka) substitutions of duplicated *BrCAMTA* genes was calculated. We used the method T = Ks/(2 × 1.5 × 10^−8^) × 10^−6^ million years ago (MYA) to estimate the divergence period for each gene pair, where Ks stands for the synonymous substitution number between two genes or sequences [50].

### 2.6. Protein Structure Modeling and PPI of *B. rapa CAMTA* Genes

The secondary structure prediction of the BrCAMTA protein sequence was conducted by SPOMA Secondary structure prediction (https://npsa-prabi.ibcp.fr/cgi-bin/npsa_ automat.pl?page=npsa_sopma.html) [51]. The tertiary structures of BrCAMTA proteins were predicted using SWISS‐MODEL (https://www.swissmodel.expasy.org) [52]. Additionally, the STRING database (https://string-db.org/) was implemented to predict potential PPIs among BrCAMTA proteins [53].

### 2.7. Investigation of Promoter Cis‐Elements in *BrCAMTA* Genes

The 1500‐bp promoter sequences of the *CAMTA* genes were retrieved from the Ensembl Plants database [31]. Cis‐acting regulatory elements (CAREs) (typically 5–10 bp in length) within the 12 BrCAMTA promoter regions were identified using the PlantCARE web‐based analysis tool [54].

### 2.8. RNA‐Sequencing Data Analysis

The expression profiles of *BrCAMTA* genes were analyzed using RNA‐sequencing data obtained from the Expression Atlas database (https://www.ebi.ac.uk/gxa/home) [55], originally developed and evaluated by Tong et al. [56]. Expression levels were assessed across six tissues: root, stem, plant callus, silique, flower, and leaves. Transcript abundance was quantified as transcripts per million (TPM). These data were subsequently used to generate a heat map of *BrCAMTA* gene expression using TBtools.

## 3. Results

### 3.1. Identification of *CAMTA* Family Genes and Sequence Analysis

A total of 12 *CAMTA* genes were identified in *B. rapa* and designated as *BrCAMTA1*–*BrCAMTA12* based on their gene IDs (Table 1). The coding sequence (CDS) lengths ranged from 729 bp in BrCAMTA5 to 3096 bp in BrCAMTA8. The predicted proteins varied in size, with BrCAMTA8 encoding the longest protein (1031 amino acids) and BrCAMTA5 the shortest (242 amino acids). Other protein lengths included BrCAMTA1 (973 aa), BrCAMTA2 (997 aa), BrCAMTA3 (1008 aa), BrCAMTA4 (263 aa), BrCAMTA6 (850 aa), BrCAMTA7 (352 aa), BrCAMTA9 (1010 aa), BrCAMTA10 (1028 aa), BrCAMTA11 (919 aa), and BrCAMTA12 (923 aa) (Table 1).

**Table 1:** Information regarding *BrCAMTA* genes and their corresponding proteins in *Brassica rapa.*

Gene name	Gene id	Chr no.	Chromosomal location			Protein length	MW^1^ (kDa)^2^	pI^3^	GRAVY^4^	ORF^5^ (bp)^6^	Subcellular localization	Domain CG‐1 (start–end)
			Start	End	Length							
BrCAMTA1	Bra004096	Chr7	16,824,046	16,828,822	22,586,724	973	108.43023	5.65	−0.579	2922	Nucleus	13–127
BrCAMTA2	Bra004217	Chr7	17,478,335	17,483,180	22,586,724	997	110.56114	6.39	−0.485	2994	Nucleus	13–132
BrCAMTA3	Bra009382	Chr10	16,410,448	16,415,231	17,594,535	1007	113.5119	5.73	−0.516	3024	Nucleus	23–137
BrCAMTA4	Bra010875	Chr8	16,887,708	16,890,485	21,594,650	263	28.58383	9.39	−0.345	792	Nucleus	187–222
BrCAMTA5	Bra016887	Chr4	17,744,437	17,745,808	18,965,343	242	27.29301	6.12	−0.238	729	Nucleus	1–44
BrCAMTA6	Bra022188	Chr5	19,207,338	19,211,720	23,939,834	850	96.75832	6.57	−0.533	2553	Nucleus	32–146
BrCAMTA7	Bra024655	Chr9	23,786,270	23,787,791	37,120,481	352	40.72991	9.03	−0.786	1059	Cell membrane, nucleus	184–274
BrCAMTA8	Bra030248	Chr4	9,635,836	9,640,798	18,965,343	1031	115.18679	5.43	−0.554	3096	Nucleus	21–135
BrCAMTA9	Bra034007	Chr2	1,025,4669	10,258,976	27,846,329	1012	113.02683	5.40	−0.607	3039	Nucleus	10–128
BrCAMTA10	Bra037769	Chr9	3,476,481	3,481,759	37,120,481	1028	115.06866	5.93	−0.448	3087	Nucleus	50–164
BrCAMTA11	Bra038040	Chr8	6,983,351	6,987,642	21,594,650	919	104.45277	7.43	0.531	2760	Nucleus	38–152
BrCAMTA12	Bra038534	Chr9	5,148,382	5,152,757	37,120,481	930	104.16051	5.50	−0.540	2793	Nucleus	21–135

*Note:* Superscripted numerals denote the following: ^1^ = molecular weight, ^2^ = kilo Dalton, ^3^ = isoelectric point, ^4^ = grand average of hydropathicity, ^5^ = open reading frame, and ^6^ = base pair.

The theoretical pI values revealed that BrCAMTA7 and BrCAMTA11 are basic proteins (pI > 7), whereas the remaining members are acidic (pI < 7). The proteins exhibited predicted MWs ranging from 27.29 kDa (BrCAMTA5) to 115.19 kDa (BrCAMTA8). The GRAVY index varied between −0.786 and 0.531, indicating that all proteins are predominantly hydrophilic, except for BrCAMTA11 (0.531), which exhibited hydrophobic characteristics. Subcellular localization analysis predicted that all BrCAMTA proteins are localized in the nucleus, consistent with their functions as nuclear TFs.

### 3.2. Phylogenetic Classification, Synteny Analysis, and Multiple Sequence Alignment of BrCAMTA Proteins

**Figure 1:**
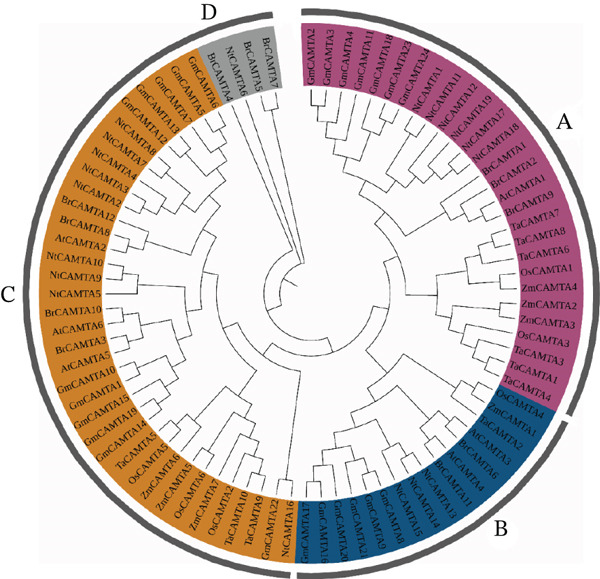
Phylogenetic analysis of CAMTA proteins from *B. rapa*, *Arabidopsis*, rice, wheat, maize, tobacco, and soybean constructed in MEGA‐12 using the neighbor‐joining method with 1000 bootstrap replicates.

A comprehensive phylogenetic analysis of CAMTA proteins was performed using 84 amino acid sequences from *B. rapa* (Chinese cabbage, 12), *A. thaliana* (Arabidopsis, 6), *O. sativa* (rice, 6), *T. aestivum* (wheat, 10), *Z. mays* (maize, 7), *N. tabacum* (tobacco, 19), and *G. max* (soybean, 24) (Figure 1). The resulting phylogenetic tree grouped the CAMTA proteins into four major clades (A–D). Among these, Group C contained the largest number of members (36), followed by Group A (28), Group B (16), and Group D (4). Except for Group D, all groups included CAMTA proteins from both monocot and dicot species (Figure 1). The BrCAMTA proteins spanned across all clades, with the majority clustering in Group C. One paralogous pair, including BrCAMTA5–BrCAMTA7, was identified, along with orthologous relationships between *B. rapa* and *A. thaliana* CAMTAs, such as BrCAMTA10–AtCAMTA6, BrCAMTA6–AtCAMTA3, BrCAMTA8–AtCAMTA2, BrCAMTA11–AtCAMTA4, and BrCAMTA3–AtCAMTA5 (Figure 1).

**Figure 2:**
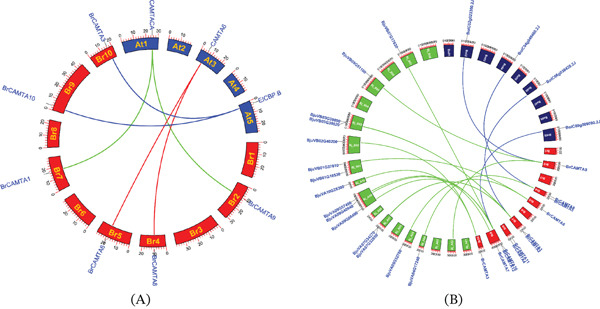
(A) Syntenic relationship between *B. rapa* and *Arabidopsis*. The *B. rapa* chromosomes are labeled from Br‐01 to Br‐10 and represented as red color; *Arabidopsis* chromosomes are labeled from At‐01 to At‐5 and represented as blue color. Red, green, and blue lines highlight the syntenic *BrCAMTA* gene pairs among two species. (B) Syntenic relationship among *B. rapa*, *B. oleracea*, and *B. juncea*. The *B. rapa* chromosomes are labeled from Br1 to Br10 and represented in red color; *B. oleracea* chromosomes are labeled from Bo01 to Bo09 and represented in blue color; *B. juncea* chromosomes are labeled from Bj_A01 to Bj_A10 and represented in lemon color. Blue lines highlight the syntenic gene pairs between *B. rapa* and *B. oleracea*, whereas lemon lines highlight the syntenic gene pairs between *B. rapa* and *B. juncea*.

We also conducted synteny analysis between *B. rapa* and *Arabidopsis*. Collinearity has been observed between the *CAMTA* genes of *B. rapa* and *A. thaliana* (Figure 2A). A total of six orthologous gene pairs have been identified. For example, *BrCAMTA1* and *BrCAMTA9* showed a syntenic relationship with *AtCAMTA1*, also known as calmodulin‐binding transcription activator protein with CG‐1 and ankyrin domain (CAMTACA) (Figure 2A). *BrCAMTA3* and *BrCAMTA10* showed a syntenic relationship with *AtCAMTA5*, also known as ethylene‐induced calmodulin binding protein (EICBP.B) (Figure 2A). *BrCAMTA6* and *BrCAMTA8* showed a syntenic relationship with *AtCAMTA3* and *AtCAMTA2*, respectively (Figure 2A). Interestingly, all of the orthologous gene pairs are closely located in the same clades of the phylogenetic tree, suggesting their functional similarity. In addition, the collinearity among *B. rapa*, *B. oleracea*, and *B. juncea* was illustrated to identify conserved collinear gene pairs across these genomes (Figure 2B). Comparative synteny analysis revealed four orthologous gene pairs between *B. rapa* and *B. oleracea*. For instance, *BrCAMTA9* and *BrCAMTA8* displayed conserved collinearity with *BolC02g023360*.2J and *BolC04g049460*.2J, respectively (Figure 2B). In contrast, 13 orthologous gene pairs were identified between *B. rapa* and *B. juncea*, indicating a higher level of genomic conservation. Among these, *BrCAMTA3* exhibited syntenic relationships with *BjuVA10G28300* and *BjuVB02G40200*, whereas *BrCAMTA6* was collinear with *BjuVA05G32760* and *BjuVB01G37810* (Figure 2B).

**Figure 3:**
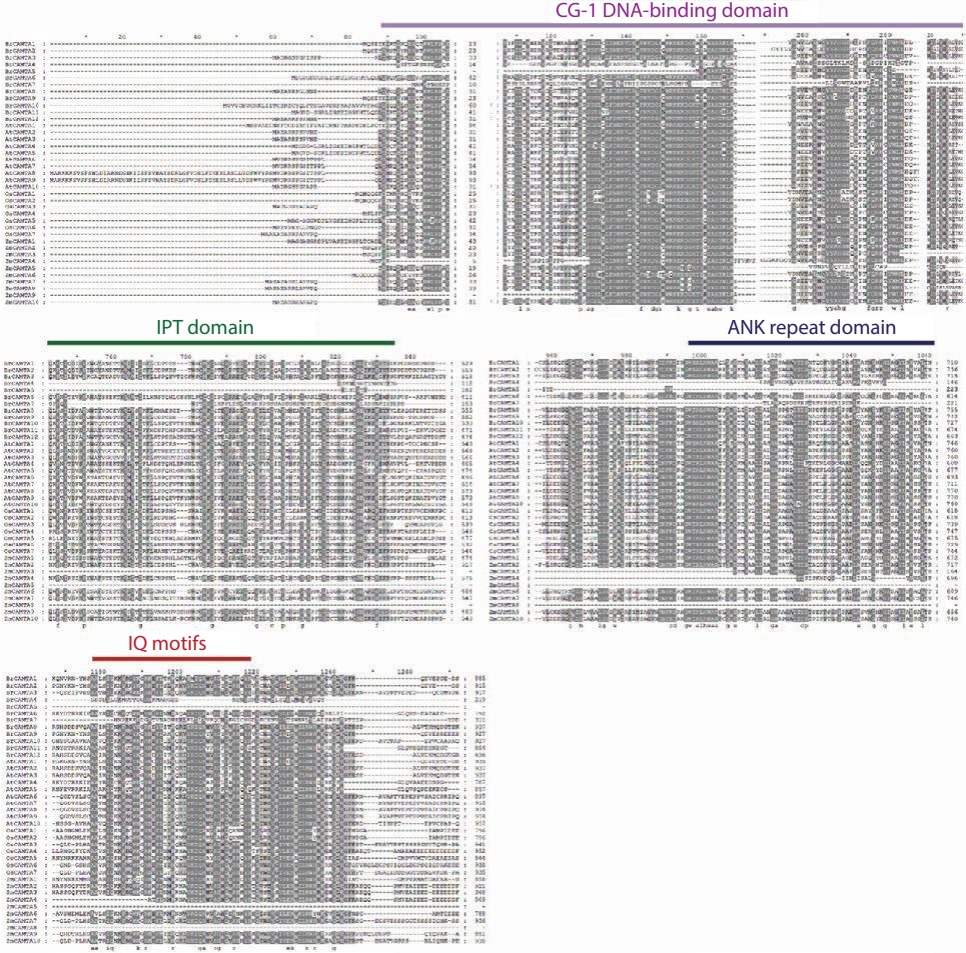
Multiple sequence alignment of BrCAMTA proteins. Conserved structural domains of the *Brassica rapa* CAMTA gene family are illustrated. Lines of different colors represent CAMTA domains: purple = CG‐1, green = IPT, blue = ANK, red = IQ; names are indicated on the figure.

Multiple sequence alignment of *B. rapa* CAMTA proteins with those from three other plant species revealed four conserved regions (Figure 3). The analysis demonstrated that CAMTA proteins share a conserved domain organization, with four key domains: CG‐1, IPT, ANK, and IQ arranged from the N‐ to C‐terminus (Figure 3).

### 3.3. Structural Assessment of *BrCAMTA* Proteins

**Figure 4:**
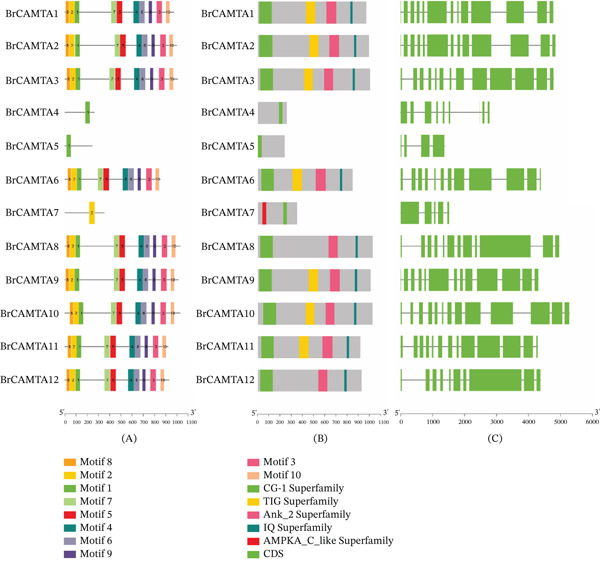
(A) Motif composition, (B) domain architecture, and (C) gene structure of *BrCAMTA* genes.

Motif analysis identified 10 conserved motifs among the BrCAMTA proteins. Except for BrCAMTA4, BrCAMTA5, and BrCAMTA7, all members contained all 10 motifs. BrCAMTA4 and BrCAMTA5 possessed only Motif 1, whereas BrCAMTA7 contained only Motif 2 (Figure 4A).

The *CAMTA* gene family is primarily characterized by the presence of a CG‐1 DNA‐binding domain, along with IQ, ANK, and TIG/IPT domains. Domain analysis revealed that all BrCAMTA members, except BrCAMTA4, BrCAMTA5, and BrCAMTA7, contained all four conserved domains, whereas these three proteins possessed only the CG‐1 superfamily domain (Figure 4B).

To gain a thorough understanding of the *CAMTA* gene evolution in *B*. *rapa*, their structural features were analyzed. The intron count per gene varied from 3 to 12, showing slight variation among different phylogenetic groups (Figures 1 and 4C). Similarly, the number of exons varied from 4 to 13 across the genes (Figure 4B).

### 3.4. Chromosomal Locations, Gene Duplication, and Calculation of Ka/Ks Ratios

**Figure 5:**
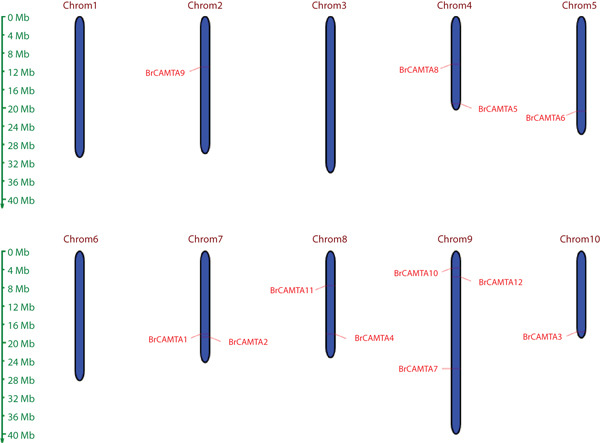
Chromosomal distribution of *BrCAMTA* genes in *B. rapa* is shown, with chromosome numbers labeled at the top of each chromosome. Chromosome lengths and gene positions were measured using the megabase (Mb) scale indicated on the left side of the figure.

The physical positions of the 12 *BrCAMTA* genes were determined by mapping them onto the 10 chromosomes of *B. rapa*. The genes exhibited an uneven chromosomal distribution. Chromosome 09 contained three genes, whereas 04, 07, and 08 each harbored two genes. Chromosomes 02 and 10 contained a single gene each, whereas no *BrCAMTA* genes were detected on 01, 03, or 06 (Figure 5).

The sequence identity ranged from as low as 0% (BrCAMTA4 and BrCAMTA7) to 90.91% (BrCAMTA4 and BrCAMTA10) (Table S1). Paralogous genes were defined as tandemly duplicated genes when they were located within 100 kb of each other on a chromosome, with no more than five intervening genes [57]. Genes exhibiting a query coverage and sequence identity of ≥ 80% were classified as segmentally duplicated genes [58]. Seven pairs of *BrCAMTA* genes were identified as segmentally duplicated, each with more than 80% sequence identity (Table S1). Phylogenetic analysis grouped these gene pairs into distinct evolutionary clusters (Figure 1). The duplicated pairs were distributed across Chromosomes 4, 8, 9, and 10, with two pairs (BrCAMTA5/BrCAMTA8 and BrCAMTA7/BrCAMTA10) located on the same chromosome and the remaining five pairs located on different chromosomes (Figure 5, Table S1). No tandem duplication events were observed in this study.

In the network of duplicated regions, the Ka/Ks ratio was calculated for seven duplicated paralogous gene pairs identified in duplication analysis. The Ka/Ks ratio can be < 1, =1, or > 1, where a value of 1 indicates neutral evolution, a ratio of < 1 reflects functional constraint under purifying (negative) selection, and a ratio of > 1 suggests accelerated evolution under positive selection [59]. All paralogous *BrCAMTA* gene pairs exhibited Ka/Ks ratios < 1, indicating that these genes are under strong purifying selection and evolving slowly at the protein level, with minor variations following duplication (Table S2). Furthermore, the seven paralogous gene pairs are estimated to have arisen through duplication events around 12 and 101 MYA (Table S2).

### 3.5. Protein Structure Modeling and PPI of *B. rapa CAMTA* Proteins

The secondary structures of BrCAMTA proteins were analyzed. The results indicated that these proteins lack *β*‐sheets, with the proportion of *α*‐helices ranging from 27.27% to 43.33%, extended chain (*β*‐strand) structures ranging from 3.26% to 11.93%, and random coils accounting for 51.76%–68.06% of the protein structures (Table S3).

**Figure 6:**
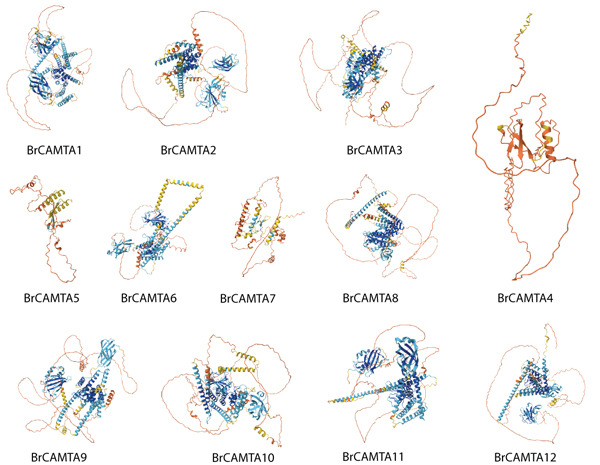
Predicted three‐dimensional structures of BrCAMTA proteins. Blue regions represent *α*‐helices.

Homology modeling was carried out for all 12 *B. rapa* CAMTA proteins to assess the accuracy of the predicted secondary structures. Three‐dimensional models were generated by selecting appropriate template structures, aligning sequences, and constructing and refining the models (Figure 6). CAMTA members within the same phylogenetic groups, specifically Group A (BrCAMTA1, BrCAMTA2, and BrCAMTA9), Group B (BrCAMTA6 and BrCAMTA11), Group C (BrCAMTA3, BrCAMTA8, BrCAMTA10, and BrCAMTA12), and exhibited similar structural features (Figures 1 and 6).

**Figure 7:**
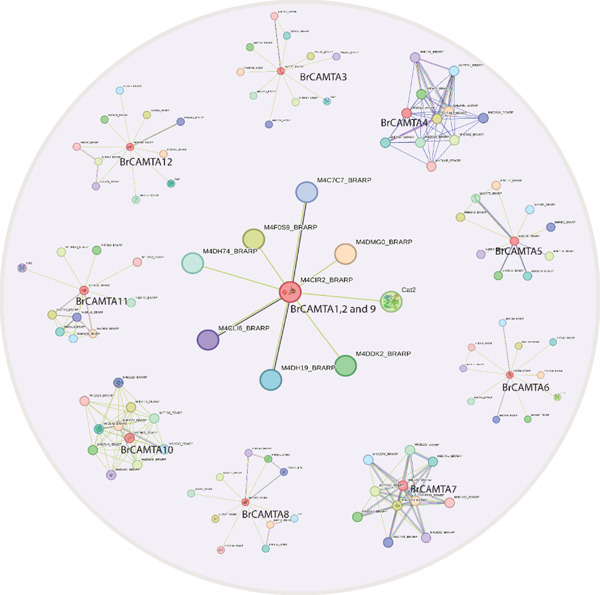
Protein–protein networking of 12 CAMTA proteins of *B. rapa.* Different colors indicate various types of interactions. Edges represent protein–protein associations.

PPI analysis is a powerful approach for investigating the potential biological roles of uncharacterized proteins. Accordingly, the BrCAMTA PPI network was predicted using a confidence score of 0.4 (Figure 7). Because the interaction setting was limited to a maximum of 10 partners per protein, each BrCAMTA showed fewer than 10 predicted interacting proteins, which are listed in detail in Table 2 (Figure 7). Among the predicted partners, several proteins such as C3H1‐type domain‐containing proteins and catalase 2 appeared frequently, with catalase 2 being known for its role in defending cells from hydrogen peroxide–induced toxicity. Other interaction partners included Nefa_Nip30_N domain‐containing proteins, AA_TRNA_LIGASE_II domain‐containing proteins, cytochrome c‐type biogenesis proteins, C3H1‐type domain‐containing proteins, ligase II domain‐containing proteins, E3 ubiquitin transferases, C2H2 and RRM domain‐containing proteins, protein kinases, bHLH proteins, chorismate mutase, hexosyltransferases, and peptidyl‐prolyl cis–trans isomerases, among others (Table 2; Figure 7).

**Table 2:** Predicted functional partner found in protein networking.

Predicted partner	Description	Gene name
M4DMG0_BRARP	Catalase acts to mitigate cellular damage caused by hydrogen peroxide	BrCAMTA1, BrCAMTA2, BrCAMTA3, BrCAMTA6, BrCAMTA9, and BrCAMTA11
M4F0S9_BRARP	Catalase acts to mitigate cellular damage caused by hydrogen peroxide	BrCAMTA1, BrCAMTA2, BrCAMTA3, BrCAMTA6, BrCAMTA9, BrCAMTA11, and BrCAMTA12
M4DH74_BRARP	C3H1‐type domain‐containing protein	BrCAMTA1, BrCAMTA2, BrCAMTA3, BrCAMTA5, BrCAMTA6, BrCAMTA9, BrCAMTA11, and BrCAMTA12
Cat2	Catalase 2	BrCAMTA1, BrCAMTA2, BrCAMTA3, BrCAMTA6, BrCAMTA9, BrCAMTA11, and BrCAMTA12
M4EYV2_BRARP	RING‐type E3 ubiquitin transferase	BrCAMTA3
M4DJY0_BRARP, M4ESK7_BRARP	AA_TRNA_LIGASE_II domain‐containing protein	BrCAMTA3
M4FH23_BRARP	Mediator of RNA polymerase II transcription subunit	BrCAMTA4
M4EHK3_BRARP	SH3 domain‐containing protein	BrCAMTA4
M4E5G5_BRARP	RRM domain‐containing protein	BrCAMTA5
M4F6P0_BRARP, M4FIP2_BRARP	C2H2‐type domain‐containing protein	BrCAMTA5
M4CH06_BRARP, M4CTX0_BRARP	Nefa_Nip30_N domain‐containing protein	BrCAMTA6 and BrCAMTA11
M4DJJ0_BRARP	Cytochrome c‐type biogenesis protein	BrCAMTA6
M4E8L9_BRARP	Component of the coenzyme Q biosynthetic pathway	BrCAMTA6
M4DCF7_BRARP, M4DED5_BRARP, M4CGZ8_BRARP	Protein kinase domain‐containing protein	BrCAMTA7
M4EIE5_BRARP	Chorismate mutase	BrCAMTA8 and BrCAMTA12
M4EVW4_BRARP, M4E8X0_BRARP M4CIY2_BRARP, M4CGB8_BRARP	BHLH domain‐containing protein	BrCAMTA8
	Belongs to the calmodulin family	
M4EEE4_BRARP (Hexosyltransferase;	Belongs to the glycosyltransferase 8 family	BrCAMTA10
M4EPU8_BRARP	Promotes the cis–trans conversion of proline imidic peptide bonds in oligopeptides	BrCAMTA11

The predicted interaction network was linked to diverse biological processes, including nutrient starvation responses, hydrogen peroxide response and catabolism, phosphorylation‐related signaling, redox homeostasis, mitochondrial seryl‐tRNA aminoacylation, reproductive development, and responses to light, metals, inorganic substances, and acidic pH (Table S4). Correspondingly, associated molecular functions encompassed oxidative stress–related activities (e.g., catalase and hydrogen peroxide catabolism), metal ion and calcium/calmodulin binding, various serine/threonine kinase activities, serine‐tRNA ligase and tRNA binding functions, phosphotransferase and ATP‐binding activities, and core promoter sequence–specific DNA binding. Together, these findings suggest that BrCAMTA interaction partners participate in wide‐ranging biochemical and regulatory pathways (Table S4).

### 3.6. Investigation of Promoter Cis‐Elements in *BrCAMTA* Genes

**Figure 8:**
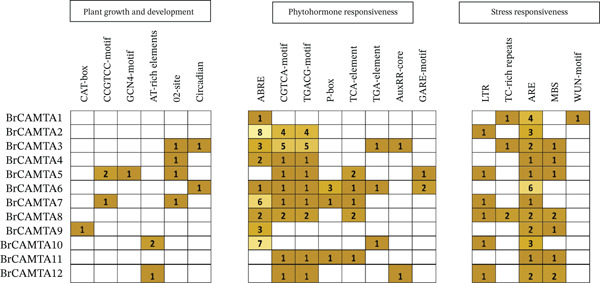
Identification of cis‐acting elements in *CAMTA* gene promoters. The column diagram displays the distribution and frequency of different CAREs in *BrCAMTA* promoters, classified into three functional categories: stress response, phytohormone signaling, and growth and development.

This analysis of cis‐acting elements strengthens our understanding of the regulatory mechanisms that may influence *BrCAMTA* genes, shedding light on their contribution to stress adaptation, development, and hormone signaling in *B. rapa*. To explore the possible biological functions of the *CAMTA* genes in *B. rapa*, we examined the cis‐acting elements within their promoters. Cis‐regulatory elements were identified using PlantCARE. Stress‐responsive elements included MBS (drought; present in eight genes), LTR (low temperature; six genes), TC‐rich repeats (defense and stress; three genes), and ARE (anaerobic conditions; 12 genes). Hormone‐responsive elements comprised ABRE (abscisic acid; nine genes), TGA (auxin; three genes), P‐box and GARE (gibberellin [GA]; three and two genes, respectively), and the CGTCA/TGACG motifs (methyl jasmonate [MeJA]; nine genes) (Figure 8). Additional cis‐elements associated with zein metabolism, circadian regulation, tissue‐specific expression, and light responsiveness were also identified (Figure 8 and Table S5). Overall, closely related *BrCAMTA* genes exhibited similar cis‐element profiles (Figures 1 and 8).

### 3.7. Expression Profiling in Different Tissues

**Figure 9:**
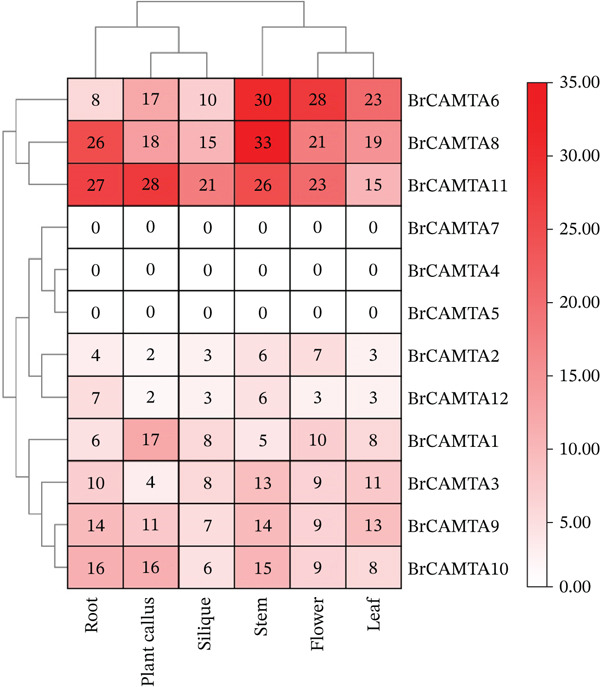
Heatmap showing expression level of *BrCAMTA* genes in different tissues. The color scale from white to red represents increasing expression levels.

RNA‐seq analysis elucidated variable expression patterns of *BrCAMTA* genes across six tissues: root, stem, callus, silique, flower, and leaf (Figure 9). *BrCAMTA6*, *BrCAMTA8*, and *BrCAMTA11* showed consistently high expression levels in all tissues, with particularly strong expression in the stem, flower, and root. *BrCAMTA1*, *BrCAMTA3*, *BrCAMTA9*, and *BrCAMTA10* exhibited moderate expression across all tissues. Specifically, *BrCAMTA6* was highly expressed in leaf and flower, *BrCAMTA8* in stem, and *BrCAMTA11* in silique, callus, and root. No expression was detected for *BrCAMTA4*, *BrCAMTA5*, and *BrCAMTA7* in any of the tissues examined (Figure 9).

## 4. Discussion

### 4.1. Overview of the *CAMTA* Gene Family

Studying plant gene families provides critical insights into how gene duplication events drive the emergence of novel functions that enhance growth, development, and stress adaptation. Such analyses also facilitate the detection of genes related to essential agricultural traits, encompassing both disease resistance and tolerance to various abiotic stresses. In plants, the ability to perceive and adapt to environmental changes is largely mediated by calcium (Ca^2+^) signals—specific fluctuations in intracellular Ca^2+^ concentrations [60]. This intricate Ca^2+^ signaling pathway involves various Ca^2+^‐binding proteins and downstream targets regulated by Ca^2+^ sensors [61, 62]. Calmodulin (CaM) is a central Ca^2+^ sensor that regulates numerous TFs in a Ca^2^‐dependent manner. *CAMTAs*, also referred to as ethylene‐induced CaM‐binding proteins or signal‐responsive proteins, are the most extensively characterized CaM‐binding TFs [2].

The well‐established U′s triangle theory posits that the three diploid species *B. rapa* (A genome), *B. nigra* (B genome), and *B. oleracea* (C genome) gave rise to the three amphidiploid species, including *B. napus*, *B. juncea*, and *B. carinata* [63]. Genetic linkage analyses have supported these relationships [61–63], and sequence homology with *Arabidopsis* has further reinforced the genetic connections across *Brassica* species [64–68]. This hybridization has led to an increased interest in utilizing *B. rapa* information and bioinformatics resources for identifying genes across the genome and examining multiple gene copies within various gene families. *CAMTA*‐mediated gene transcription control plays a role in regulating plant responses to hormones and environmental stresses [13, 22, 69–71]. *CAMTA* TFs are crucial mediators of Ca^2+^/CaM signaling [8].

*CAMTA* gene family members have been identified in various eukaryotes, especially in model plants such as *Arabidopsis*, rice, wheat, and maize [12, 19]. These genes play critical roles in regulating plant hormone signaling and abiotic stress responses. However, until now, a genome‐wide analysis of *CAMTA* genes had not been conducted in *B. rapa*. Given the agricultural significance of *Brassica* species and their complex polyploidy history, this study is aimed at filling that gap.

### 4.2. Physicochemical Properties

We identified 12 *BrCAMTA* genes in *B. rapa*. Analysis of physicochemical properties revealed variation in protein length, pI, MW, and hydrophilicity among the *BrCAMTA* proteins, indicating a wide range of potential functional diversity within the family. Subcellular localization analysis indicated that most *BrCAMTA* proteins are predominantly localized in the nucleus, consistent with their function as TFs. Notably, *BrCAMTA7* was additionally predicted to localize to the plasma membrane (Table 1).

### 4.3. Phylogenetic Classification and Synteny Analysis

Our phylogenetic analysis revealed that BrCAMTA proteins are more closely related to AtCAMTA proteins than to CAMTA proteins from other crop species, suggesting a shared evolutionary origin and potentially conserved functions. The analysis grouped 84 CAMTA proteins into four major clades, with all 12 BrCAMTAs represented across these. The presence of both monocot and dicot species in Groups A, B, and C suggests that *CAMTA* gene diversification likely occurred before the monocot–dicot split, whereas Group D contains only dicot members. Observed paralogous and orthologous relationships point to both ancient gene duplication events and functional conservation. Paralogs are homologous genes that arise within a species via duplication and may acquire new but related functions [72], whereas orthologs result from speciation events and typically retain the function of their ancestral gene [72]. Thus, the intergenomic connections among *B. rapa*, *B. oleracea*, and *B. juncea* indicate a common ancestral origin and the conservation of genomic blocks during speciation events throughout *Brassica* genome evolution (Figure 2B). The higher degree of collinearity observed between *B. rapa* (AA) and *B. juncea* (AABB) can be attributed to the allotetraploid nature of *B. juncea*, which contains the A genome derived from *B. rapa*. This conserved synteny reflects their close evolutionary relationship, consistent with the U′s triangle model [73].

### 4.4. Structural Assessment of *BrCAMTA* Genes

Functional conservation is also inferred from *Arabidopsis* orthologous. The comparative synteny analysis of *B. rapa* alongside *A. thaliana*, a dicot species, further emphasizes the potential evolutionary processes involved in the *BrCAMTA* gene family (Figure 2). For example, BrCAMTA8 and BrCAMTA6, which are grouped with AtCAMTA2 and AtCAMTA3, may play roles in cold and drought stress tolerance, pathogen resistance, and herbivore defense [8, 20, 22, 71, 74–76].

Similar gene structures and motif patterns within groups support this evolutionary link. Motif analysis using MEME showed that *CAMTA* genes within the same phylogenetic group shared similar motif compositions, suggesting that gene family expansion may have occurred through duplication of a common ancestor (Figure 4A). Variations in motif presence between groups also point to functional diversification over time.

Gene structure analysis revealed that most *BrCAMTA* genes contained 11–12 introns. The intron composition of *CAMTA* genes varies across plant species. For instance, papaya possesses two *CAMTA* genes containing 6 and 13 introns, respectively [77]. These findings align with intron counts reported in other species: 10–13 in wheat [28], 9–12 in *Gossypium* [76], 6–12 in citrus [78], and 10–12 in banana [79] (Figure 4C). The high intron conservation indicates evolutionary stability.

### 4.5. Gene Duplication and Calculation of Ka/Ks Ratios

Gene family expansion is predominantly driven by duplication events, including both segmental and tandem duplications. In particular, tandem duplications have been implicated in enhancing plant resilience to various environmental stresses, thereby contributing to adaptive evolution [80]. In our study, no tandem duplication events were observed within the *BrCAMTA* gene family; however, seven segmental duplication events were identified (Table S1). This suggests that segmental duplication may be the primary mechanism driving the expansion of *BrCAMTA* genes in *B. rapa*. This pattern reflects the evolutionary history of the *Brassica* lineage and a whole‐genome triplication event after it diverged from *A. thaliana* [68]. This polyploidization event generated numerous paralogous chromosomal blocks that followed distinct scenarios of gene retention, loss, and functional divergence. The presence of several segmentally duplicated *BrCAMTA* gene pairs likely reflects the retention of genes derived from these triplicated genomic regions. Such retention is often influenced by dosage‐balance constraints, particularly for regulatory genes such as TFs, which tend to be preferentially preserved following whole‐genome duplication events [81]. Similar observations have been made in other species, including banana [79], and *Cucurbita moschata* and *Cucurbita maxima* [24], where most identified *CAMTA* genes were mapped to various chromosomes.

The Ka/Ks ratio helps identify selection pressure. In the current study, duplicated *BrCAMTA* gene pairs showed Ka/Ks values of less than 1, suggesting that most duplicated *BrCAMTA* genes were primarily subjected to purifying selection after genomic duplication. This evolutionary constraint indicates that *BrCAMTA* genes preserve fundamental biological functions, aligning with previously published reports emphasizing essential roles for *CAMTA* TFs in plant stress signaling pathways [22]. This pattern of functional conservation is also widely reported among the duplicate genes retained after whole‐genome duplication events in Brassica species descended from a triplicated common ancestor genome [82, 83].

In addition, seven paralogous genes were found to have undergone segmental duplication in this study (Table S2). We calculated that the *BrCAMTA* genes′ segmental duplication event happened between 29.90 and 65.09 MYA (Table S2). It is possible that *Brassica* and *Arabidopsis* broke apart between 23.4 and 33.5 MYA [84]. As a result, *BrCAMTA* gene duplication regularly happens during *B. rapa*′s evolution both before and after the species separated from the *Arabidopsis* lineage.

### 4.6. Protein Structure Modeling and PPI

The capacity of amino acid residues to form hydrogen bonds and their hydrophobicity were used to predict the secondary structure of the CAMTA protein, which includes the distribution and percentage of the *α*‐helix, *β*‐angle, and random curling. The absence of *β*‐angles suggests that some peptide chains lack *β*‐structure formation or have a low fraction of angles, making them “0” or nonexistent.

A study of the complex network of protein interactions resulted in the discovery of a novel protein family that plays a vital role in plant growth, particularly plant meristem and leaf formation [85]. We found that BrCAMTA proteins primarily interact with proteins containing C2H2, C3H1, SH3, RRM, Nefa_Nip30_N, and BHLH domains, members of the calmodulin protein family, as well as various catalases (Table 2, Figure 7). The function of catalase (CAT2) in drought and cold stress, whereas CAT3 was related to abscisic acid and oxidative stress, which is found in *Arabidopsis* [86]. Furthermore, catalase 2 appears to be involved in a diverse array of metabolic and signaling networks, as it was found to be associated with pathways such as tryptophan metabolism, glyoxylate and dicarboxylate metabolism, carbon metabolism, the biosynthesis of secondary metabolites, and peroxisomal function. Notably, its predicted involvement in the plant mitogen‐activated protein kinase (MAPK) signaling pathway warrants a closer examination of this cascade. MAPK cascades are evolutionarily conserved signaling modules in eukaryotes that transduce extracellular signals to the nucleus or cytoplasm, eliciting appropriate cellular responses, including cell division, differentiation, programmed cell death, and stress adaptation. In plants, these cascades are triggered by various biotic and abiotic stimuli such as pathogen infection, wounding, low temperature, drought, osmotic shock, high salinity, and reactive oxygen species. Beyond stress responses, MAPK pathways are also integral to hormonal and developmental signaling. A key feature of these networks is their ability to maintain signaling specificity even when multiple pathways, initiated from distinct receptors, share common kinase components. It is important to note that, unlike those in animals and fungi, all plant MAPK genes characterized to date belong to the extracellular signal‐regulated kinase (ERK) subfamily (KEGG Pathway: brp04016). Besides catalases, the other interacting proteins are involved in cell structure, signal transduction, and responses to both abiotic and biotic stresses.

### 4.7. Investigation of Promoter Cis‐Elements in *BrCAMTA* Genes

CAREs in promoter regions govern gene expression under various environmental and developmental conditions. Investigating these elements is vital for gaining clearer insights into gene regulation processes [87]. The *BrCAMTA* promoters were enriched with elements responsive to drought (MBS), defense (TC‐rich repeats), low temperature (LTR), anaerobic conditions (ARE), and several hormones such as abscisic acid (ABRE), GA (P‐box, GARE), auxin (TGA), and MeJA (CGTCA/TGACG) [88, 89] (Figure 7). Most *BrCAMTAs* contained these CAREs, further supporting their potential involvement in stress and hormone signaling (Figure 7). Since ABA induces changes in actin organization that regulate stomatal closure during stress responses and wound healing, the widespread ABA responsiveness highlights the diverse role of *BrCAMTA* genes in stress adaptation [90, 91]. Previous research has indicated that hormones like GAs and MeJA play roles in regulating fruit firmness. GA delays maturation through auxin upregulation [92], whereas MeJA facilitates fruit softening by enhancing *XTH1* and *EG1* expression [93].

### 4.8. Expression Profiling in Different Tissues

Differential expression of genes across tissues can provide insights into their functional diversity. The varying expression levels of *BrCAMTA* genes in different tissues suggest that functional diversification may have occurred during evolution and indicate that these genes likely play distinct regulatory roles in *B. rapa* development. *BrCAMTA6*, *BrCAMTA8*, and *BrCAMTA11* exhibited consistently high expression across all examined tissues (Figure 9), suggesting that they may act as key regulators in leaf morphogenesis, root architecture, and nutrient/water acquisition, as well as in floral development, potentially through coordinated interaction with flower‐specific transcriptional networks. Similar expression trends have been reported in tomato, where *CAMTA* genes are highly expressed in root and shoot tissues [7].

*CmaCAMTA1–6* showed elevated expression in fruit relative to leaf, suggesting that *CmaCAMTAs* play a role in mediating fruit development [24]. The differential gene expression analysis provided insights into functional diversity, highlighting *OsCAMTA3b* as the most highly upregulated stress‐responsive gene [4]. Compared with wild‐type *Arabidopsis*, *TgCAMTA1*/*3*‐overexpressing lines displayed significantly enhanced tolerance to freezing stress. This improved stress resilience was associated with elevated expression of *AtCOR* genes, suggesting that *TgCAMTA1*/*3* positively regulates cold‐responsive pathways to confer freezing tolerance [94]. *ZmCAMTA1* and *ZmCAMTA2* showed elevated expression throughout all stages of development, suggesting their involvement in growth and developmental processes [95].

### 4.9. Future Directions and *Brassica* Improvement

The findings from this genome‐wide analysis of the *BrCAMTA* gene family provide a strong foundation for future applications aimed at improving *Brassica* crops. The predicted interaction network generated in this study offers a roadmap for future functional genomics efforts. The diverse partners identified here should be prioritized for experimental validation using orthogonal methods such as yeast two‐hybrid or coimmunoprecipitation. Integrating these validated interactions with known pathway contexts will be essential to fully comprehend the biological relevance of BrCAMTA‐mediated protein assemblies.

The promoter analysis revealed several cis‐elements associated with stress responsiveness, suggesting that *CAMTA* genes may play diverse roles in stress adaptation. These findings provide a foundation for future experimental validation. Specifically, expression profiling via qRT‐PCR using tissues subjected to key abiotic (e.g., drought, cold, and salinity) and/or biotic (e.g., pathogen infection) stresses could be performed. Correlating these expression patterns with stress tolerance phenotypes would further clarify the functional roles of *CAMTA* genes in stress adaptation.

Despite the high expression levels of *BrCAMTA6*, *BrCAMTA8*, and *BrCAMTA11* in roots, stems, and callus tissues (Figure 9), this study did not directly test their association with quantifiable agronomic traits. This highlights the need for future studies to validate their biological functions.

• Root expression: *BrCAMTA6*, *BrCAMTA8*, and *BrCAMTA11* may influence root system architecture, affecting primary root elongation, lateral root density, and biomass accumulation under abiotic stresses such as drought or salinity.
• Stem expression: High expression in stems could be linked to vegetative growth and overall biomass production.
• Callus expression: Elevated expression in callus may be associated with cell proliferation and regeneration processes.

Future work could combine expression analysis with phenotypic evaluation across diverse *B. rapa* germplasm under control and stress conditions. Additionally, expression levels of *BrCAMTA* genes or haplotype variation could be correlated with traits such as root architecture, biomass, stress tolerance indices, and physiological responses. Functional validation may be further pursued via CRISPR/Cas9‐mediated gene knockout, overexpression studies, or association mapping to determine whether allelic variation in BrCAMTA genes contributes to important agronomic traits.

The identification of key candidates, *BrCAMTA6*, *BrCAMTA8*, and *BrCAMTA11*, creates new opportunities for targeted functional studies and advanced breeding efforts. These genes represent promising targets for CRISPR/Cas‐mediated genome editing to develop stress‐resilient lines, as well as for transgenic overexpression or knockout approaches to clarify their specific roles in growth and stress response pathways. In addition, they hold significant potential for integration into marker‐assisted selection and genomic selection pipelines, enabling the accelerated development of improved *Brassica* cultivars.

## 5. Conclusions

In this study, we identified and thoroughly characterized 12 *CAMTA* genes in *B. rapa*, providing a detailed analysis of their structural, evolutionary, and functional attributes. Phylogenetic analysis and sequence alignment revealed conserved evolutionary relationships, whereas gene structure and motif assessments highlighted both shared and distinct features among family members. Chromosomal localization and duplication analyses indicated that the expansion of the *BrCAMTA* gene family has been driven by segmental duplication events. Analysis of promoters identified numerous cis‐regulatory elements related to hormonal and stress responses, and tissue‐specific expression patterns suggest that *BrCAMTA* genes are involved in multiple aspects of plant development and environmental adaptation. Collectively, these findings lay a strong foundation for future functional studies of *CAMTA* TFs in *B. rapa* and furnish a key understanding of their potential utility in improving stress resilience and productivity in *Brassica* crops.

**Nomenclature**
ANK: ankyrin repeats
BLAST: Basic Local Alignment Search Tool
CAMTA: calmodulin‐binding transcription activators
CDS: coding DNA sequences
CG‐1: calmodulin‐binding transcription activator 1
GRAVY: grand average of hydropathicity
HMM: hidden Markov model
kDa: kilo Dalton
Ml. Wt: molecular weight
NCBI: National Center for Biotechnology Information
pI: isoelectric point
SMART: Simple Modular Architecture Research Tool
TFs: transcription factors
TIG: transcription factor immunoglobulin
TPM: transcripts per million
UTRs: untranslated regions

## Author Contributions

Jannatul Afrin: data curation, formal analysis, investigation, and writing—original draft. Maria Chowdhory: data curation, formal analysis, visualization, and writing—original draft. Subroto Das Jyoti: data curation, formal analysis, and writing—review. Jaber Bin Azim: formal analysis and writing—review. Nikunjo Chakroborty: formal analysis. Arif Hasan Khan Robin: conceptualization, supervision, and writing—review & editing. Jannatul Afrin and Maria Chowdhory equally contributed to this manuscript.

## Funding

This study was supported by Bangladesh Agricultural University Research System (10.13039/100019278) BAURES Project No. 2023/110/BAU.

## Ethics Statement

This article does not contain any studies with human or animal subjects.

## Conflicts of Interest

The authors declare no conflicts of interest.

## Conflicts of Interest

The authors declare no conflicts of interest.

## Supporting Information

Additional supporting information can be found online in the Supporting Information section.

## Supporting information


**Supporting Information 1** Data File S1: Protein sequences of *Brassica rapa*, *Arabidopsis*, rice, bread wheat, maize, cabbage, and radish used in in silico analysis. Data File S2: Coding sequences (CDS) of *Brassica rapa* used in in silico analysis. Data File S3: Genome sequences of *Brassica rapa* used in in silico analysis.

**Supporting Information 2** Table S1: Sequence identity (%) among the 12 CAMTA proteins of *B. rapa*. Table S2: *BrCAMTA* family gene duplication event time in *B. rapa*. Table S3: Secondary structure prediction of BrCAMTA protein. Table S4: Gene ontology (GO)–based functional prediction of *CAMTA* genes. Table S5: Detailed information about cis‐acting elements identified from PlantCARE database.

## Data Availability

The data that supports the findings of this study are available in the supporting information of this article.
